# The Influence of Pelvic Tilt on the Anteversion Angle of the Acetabular Prosthesis

**DOI:** 10.1111/os.12543

**Published:** 2019-10-29

**Authors:** Guoyue Yang, Yayue Li, Hong Zhang

**Affiliations:** ^1^ Orthopaedic Department Third Central Hospital of Tianjin Tianjin China; ^2^ Tianjin Institute of Hepatobiliary Disease Tianjin China; ^3^ Tianjin Key Laboratory of Artificial Cell Tianjin China; ^4^ Artificial Cell Engineering Technology Research Center of Public Health Ministry Tianjin China; ^5^ Orthopaedic Department The Fourth Medical Center of the Chinese People's Liberation Army General Hospital Beijing China

**Keywords:** Pelvic kinematics, Pelvic tilt, Planning, Spine hip syndrome, Total hip arthroplasty

## Abstract

The concept of the “safe area” of the acetabular prosthesis has a long history and has been recognized by many scholars. It is generally believed that postoperative hip dislocation rate is low, when the acetabular anteversion angle is placed in the range of 15° ± 10°. Despite this, hip dislocation is a common complication after total hip arthroplasty. In recent years, more and more scholars have paid attention to the influence of pelvic tilt on the acetabular anteversion angle. The concept of acetabular anteversion changes as the pelvic tilt changes, and is challenging the traditional acetabular prosthesis “safe area.” This study summarized the potential influencing factors of pelvic tilt and discussed the influence of the phenomenon on the anteversion angle of total hip arthroplasty (THA) acetabular prosthesis based on the literature review. We conclude that from the supine position to standing, followed by sitting, the pelvis tends to move backward. Pelvic sagittal activity, lumbar disease (ankylosing spondylitis), lumbar fusion (lumbar fusion, spine‐pelvic fusion), and other factors related to the tilt are THA risk factors for postoperative dislocation and revision. With the change of body position, the degree of acetabular anteversion is directly related to the degree of pelvic tilt. The acetabular anteversion varies greatly, which leads to increased hip prosthesis wear and even hip dislocation. The lateral X‐ray of the spine and pelvis is recommended in supine, standing, and sitting positions before THA. In addition, the pelvic tilt should be regarded as a reference of the acetabular prosthesis in the preoperative planning of THA.

## Introduction

Upright walking is one of the signs that differentiate humans from other primates. The human body is symmetrical in the coronal plane, and the mechanical balance of the sagittal plane is also guaranteed. The sagittal morphology of the spine and pelvic tilt are coordinated to maintain balance of the limbs and for stress transmission^1^, ^2^, ^3^, ^4^. Reportedly, the sagittal deformity of the spine often needs to be compensated by changing the pelvic tilt to achieve a new mechanical balance^5^, ^6^. In addition, hip joint diseases alter the pelvic tilt, which also affects the sagittal morphology of the spine. Offierski *et al*.^7^ defined this phenomenon as a hip‐spine syndrome (HSS). Simultaneously, some studies suggested that changes in pelvic tilt would lead to changes in the acetabular anteversion^8^, ^9^.This acetabular anteversion is essential for the stability of prosthesis and the rate of long‐term wear after THA. The acetabular anteversion alters corresponding to the pelvic tilt changes, which challenges the concept of the “safe zone” of acetabular prosthesis. It is determined in the surgical position (lateral or supine position) that might not be an appropriate functional position (such as standing and sitting positions), which is caused by the stability and service life of the postoperative prosthesis. The present study aimed to investigate the influencing factors of the sagittal pelvic tilt and the effects of pelvic tilt on the outcomes of THA.

## Sagittal Spinopelvic Parameters and the Reference Values

**Figure 1:**
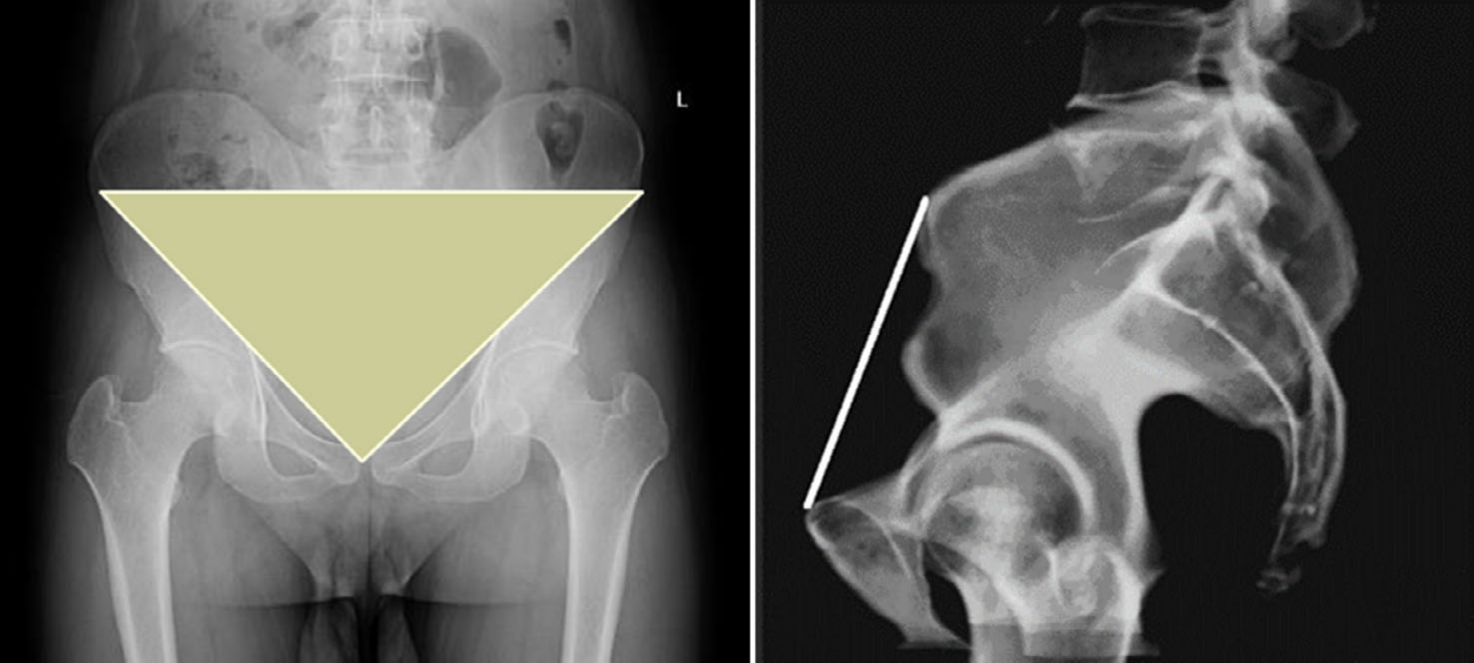
APP: The plane formed by the anterior superior iliac spine and pubic symphysis.

In recent years, many scholars have paid attention to the concept of pelvic tilt. In 1990, Anda *et al*.^9^ proposed the concept of the anterior pelvic plane (APP), which was defined as the plane formed by the bilateral anterior superior iliac spines and the upper margin of pubic symphysis. The angle between the APP and the vertical line is used to evaluate the degree of the pelvic tilt and is known as the APP angle. The formed angle in the front of the vertical line is considered positive, while it is negative if the formed angle is at the rear of the vertical line. The angle is ‐6° on average for males, while it is ‐4.3° for females (Fig. 1).

**Figure 2:**
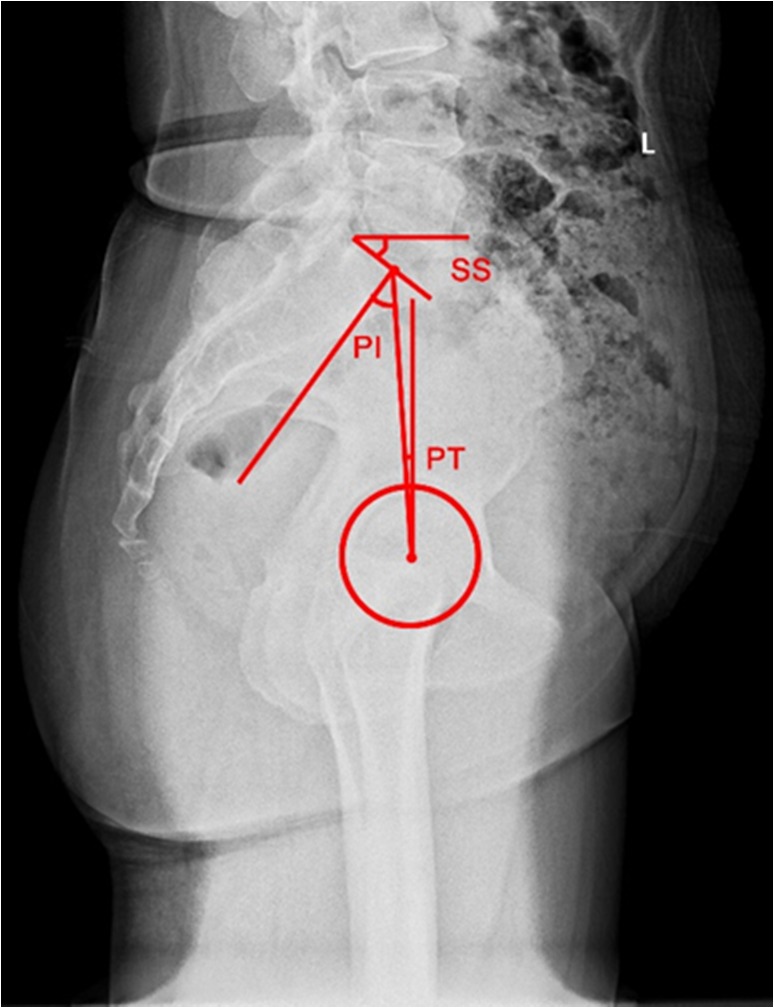
PI: The angle between the line connecting the midpoint of the S_1_ endplate to the center of the femoral head and the vertical line. The midpoint of the center line of the bilateral femoral heads is used if bilateral femoral heads do not overlap. PI is positive if the midpoint of the S_1_ endplate is at the rear of the femoral head, otherwise, it is negative. PT: The angle between the line connecting the midpoint of the S_1_ endplate to the center of the femoral head and the vertical line. SS: The angle between the tangent of the S_1_ endplate and the horizontal line.

**Figure 3:**
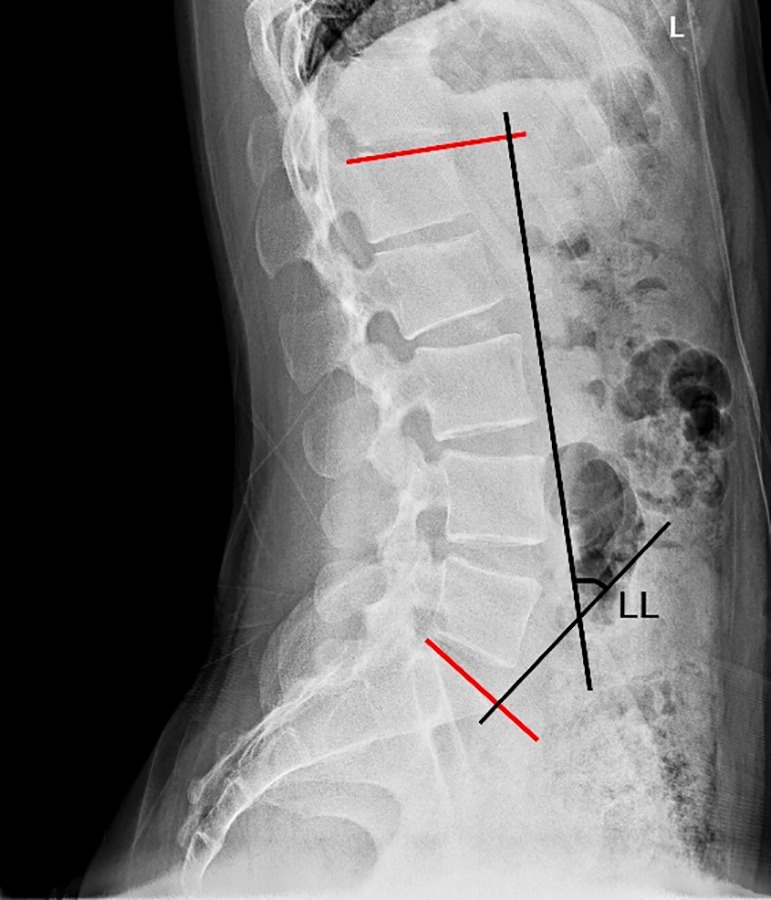
LL: The angle between the tangent of L_1_ and S_1_ endplate.

The pelvis is the platform that connects the spine to the lower limbs. The pelvic tilt is not an isolated parameter and is closely related to the sagittal morphology of the spine. Duval‐Beaupere *et al*.^10^, for the first time, proposed the concept of pelvic incidence (PI); the angle is between the line connecting the midpoint of S_1_ endplate to the center of the femoral head and the vertical line of S_1_ endplate. The PI is determined by the positions of the sacrum and femoral head, which is an anatomical parameter. Before maturity, PI increases with age, and after maturity, it is constant and not affect by the body's position^11^. While measuring the PI using the lateral pelvic radiograph, the midpoint of the center line of the bilateral femoral heads is considered if the bilateral femoral heads do not overlap (Fig. 2). In addition, the sagittal spinopelvic parameters include the following^12^: pelvic tilt (PT): the angle between the line connecting the midpoint of the S_1_ endplate to the center of the femoral head and the vertical line; and sacral slope (SS): the angle between the tangent of the S_1_ endplate and the horizontal line (Fig. 2). Both PT and SS are the positional parameters of the pelvis, which alter with changes in the pelvic position. The spinopelvic parameters obey the geometrical rule of PI = SS + PT. PI determines the direction of the pelvis while standing, and patients with large PI tend to have a large SS and/or PT. When PI is fixed and if the pelvis leans forward, PT becomes small while SS becomes large. If the pelvis leans backward, the PT becomes large while the SS becomes small^13^. The most common measurement for lumbar lordosis (LL) is Cobb's method, which refers to the angle between the tangent of the L_1_ and S_1_ endplates^14^ (Fig. 3).

The literature reported differences in the normal values of pelvic tilt parameters. Jacobsen *et al*.^15^ statistically analyzed the lateral lumbar radiograph in the standing in normal population and used SS to evaluate the pelvic tilt. The SS was speculated to be 38° on average and defined as the median pelvis. In 2004, Mac‐Thiong *et al*.^11^ reported sagittal spinopelvic parameters for children and adolescents, 4–18 years of age: PI is 48.4°, SS is 41.2°, PT is 7.2°, and LL is 48.5°. Furthermore, in 2010, the group^16^ reported the sagittal spinopelvic parameters of normal adults aged 18–81 years as follows: PI is 52.7°, SS is 39.3°, and PT is 13.4° for males, while PI is 52.4°, SS is 39.8°, and PT is 12.7° for females. Roussouly *et al*.^17^ reported the sagittal spinopelvic parameters for 160 normal adults, aged 18–48 years: PI is 52°, SS is 40°, and PT is 12°. A systematic review by Kuntz *et al*.^18^ recruited adult volunteers without spinal diseases as subjects and from a total of 12 references. The study speculated that in the 95% confidence interval, the SS of the normal adults is 20–65°, PT is 1°–25°, and LL (T12‐S1) is 40°–84°. Liu *et al*.^19^ measured the sagittal spinopelvic parameters of 40 normal Chinese adults (23 men and 17 women, 20–65 years), and the results demonstrated that PI is 47.7°, SS is 32.2°, and PT is 14.9°. Han *et al*.^20^ measured the sagittal spinopelvic parameters of 100 normal young adults in China (69 men and 31 women, 18–39 years), and revealed that the LL is 50.8°, PI is 47.2°, PT is 9.6°, and SS is 37.5°. Another study demonstrated the differences in pelvic development among human races that are manifested as diverse reference values of PI^21^.

## Correlation between Sagittal Morphology of the Spine and Pelvic Tilt

The sagittal spinopelvic balance is achieved by PI, SS, and LL^22^. Interestingly, statistical correlations were established between PI and SS, SS and LL, and PI and LL^22^, ^23^. LL was proposed to be directly affected by SS, and the two were positively correlated. The changes in the pelvic tilt need to be compensated by changes in the LL, eventually reaching the balance of the sagittal plane^24^, ^25^. Different compensation mechanisms might be activated when the spine is deformed: the spinal segments on both sides of the deformed parts can be compensated in the case of adequate spinal flexibility. When the spine is stiff, the only way of compensation is to change the pelvic tilt. For instance, when lumbar degeneration shows a flat waist deformity, LL decreases, SS compensatory decreases, and the pelvis leans backward^26^.

## Effects of Pelvic Tilt on Acetabular Anteversion

The literature reports that the relationship between pelvic tilt and acetabular anteversion has a certain regularity. Buckland *et al*.^8^.demonstrated that the change in anteversion and pelvic tilt is close to 1:1. Anda *et al*.^9^ pointed out that every 1° change in the pelvic tilt would lead to a 0.5° change in acetabular anteversion. Furthermore, the study by Dorr *et al*.^27^ showed that every 1° increase in anterior pelvic tilt would result in a reduction of acetabular anteversion by 0.7°–0.8°. Other studies also obtained similar conclusions^24^, ^25^, ^28^.

## Effects of Spinal Diseases on THA

LL is reported to be significantly correlated with pelvic tilt. If LL increases, then the pelvis is tilted forward, whereas, if it decreases, the pelvis is tilted backward^29^. The study by DelSole *et al*. revealed that lumbar back deformity and the decompensated state of the sagittal plane of the spine affect the stability of the prosthesis after THA^30^. The sagittal deformity caused by spinal diseases is compensated by the change in the pelvic tilt to achieve the sagittal balance of the limbs. Eventually, this change affects the anteversion in acetabular prosthesis during THA.

Lumbar fusion is a risk factor for dislocation and revision after THA. An *et al*.^31^ conducted a meta‐analysis based on the effects of lumbar fusion on the dislocation and revision of THA, which included six references with a total of 1,456,898 patients. In the study, 26,411 patients had a history of lumbar fusion before THA, while 1,430,387 patients did not present any such history of lumbar fusion before THA. Furthermore, the comparison between the two groups revealed a significant increase in the revision rate after THA in the group with a history of lumbar fusion. Perfetti *et al*.^32^ included a total of 934 patients who underwent THA and had a history of lumbar fusion, while another 934 patients underwent THA and did not present any history of lumbar fusion. The comparison showed a statistically significant difference *P* < 0.01 in the rate of dislocation at 12 months after THA, which is 3% for patients with a history of lumbar fusion and 0.4% for patients without the history of lumbar fusion. The study by Sing *et al*.^33^ demonstrated that, as compared to simple THA for the first time, postoperative dislocation, revision, prosthesis loosening, and other complications increase significantly in patients with a history of lumbar fusion to undergo THA. In summary, the history of lumbar fusion is a risk factor for dislocation and revision after THA.

Spinal‐pelvic fusion after THA will affect pelvic tilt and acetabular anteversion. Mudrick *et al*.^34^ reported that one case underwent THA due to hip osteoarthritis after lumbar fusion of 3–5 vertebrae. The patients suffered from adjacent segment degeneration in lumbar spine 2 years after the operation; thus, T_I0_‐S_1_ fusion is performed. Two months after the operation, the anterior dislocation of the hip joint occurs, the prosthesis is still unstable after closed reduction, and dislocation occurs three times within 2 months. After excluding the problem of soft tissue tension, the SS changes from 17° before spinopelvic fusion to 29° after the operation, while the acetabular anteversion changes from preoperative 31° to postoperative 21°. In the hip revision surgery, the anterior tilt of femoral prosthesis is satisfactory, the dislocation occurs if the lower extremity rotates 5° internally, and the acetabular prosthesis is removed. The anteversion in the reconstructed acetabular prosthesis increases by an additional 20° as compared to the primary hip replacement, and bipolar prosthesis is used. The dislocation does not occur within the postoperative 6 months. Buckland *et al*.^35^ speculated that sagittal orthopaedic surgery of the spine performed after THA causes the pelvis to lean forward and the acetabular anteversion becomes small, thereby affecting the stability of the rear part of the hip prosthesis.

Abnormal spine‐pelvic sagittal morphology is a cause of dislocation of patients with ankylosing spondylitis after THA. The pelvis is usually tilted backward extremely for patients with ankylosing spondylitis in the standing position due to malformation of the sagittal plane of the spine (thoracic kyphosis increases and lumbar lordosis decreases)^36^, ^37^, ^38^, ^39^. Interestingly, the acetabular anteversion increases as the pelvis is tilted backward^11^, ^31^, ^32^. In 2000, Tang *et al*.^40^ reported 58 patients with ankylosing spondylitis who underwent THA. After the operation, nine cases presented an acetabular revision. The study speculated that the primary cause of postoperative dislocation and revision is the excessive backward extension of the hip joint in the standing position. Furthermore, in 2007, Tang *et al*.^41^ stimulated the posterior pelvic tilt through 3D CT. The placement direction of acetabular prosthesis was adjusted based on the posterior pelvic tilt. The abduction angle and anteversion were reduced to prevent the anterior dislocation after the operation. This method increases the stability of the hip joint in the standing position but reduces the bone‐implant contact area of acetabular prosthesis. Blizzard *et al*.^42^ proposed an impact on the rear, instability in the front part, as well as anterior dislocation in acetabular prosthesis and femoral prosthesis for patients with ankylosing spondylitis and severe sagittal spinal deformity attributed to extremely large anteversion in acetabular prosthesis during THA. The patients with ankylosing spondylitis were recommended sagittal spinopelvic balance in THA preoperative planning; the angle of the acetabular prosthesis should be adjusted to adapt to the excessive posterior pelvic tilt in the standing position. The study by Zheng *et al*. enrolled a total of 28 patients with ankylosing spondylitis who require THA and spinal orthopaedic surgery. The first group included 22 patients who underwent spinal orthopaedic surgery firstly, while the second group included six cases with severe spinal deformity who received THA firstly. In the second group, two cases had dislocation of the hip joint in the early stage after the operation, and passive posture fixation was performed after closed reduction, followed by spinal osteotomy at 2 weeks after the operation. Any dislocation was not recorded in either of the two groups during the follow‐up. Thus, spinal correction surgery should be performed firstly for patients with ankylosing spondylitis who require spinal correction and THA. If the spinal or hip deformity is extremely severe, THA can be performed first with an appropriate angle of acetabular prosthesis design to avoid anterior dislocation of the hip due to excessive anterior pelvic tilt^43^.

## Effects of Body Position on the Pelvic Tilt

**Figure 4:**
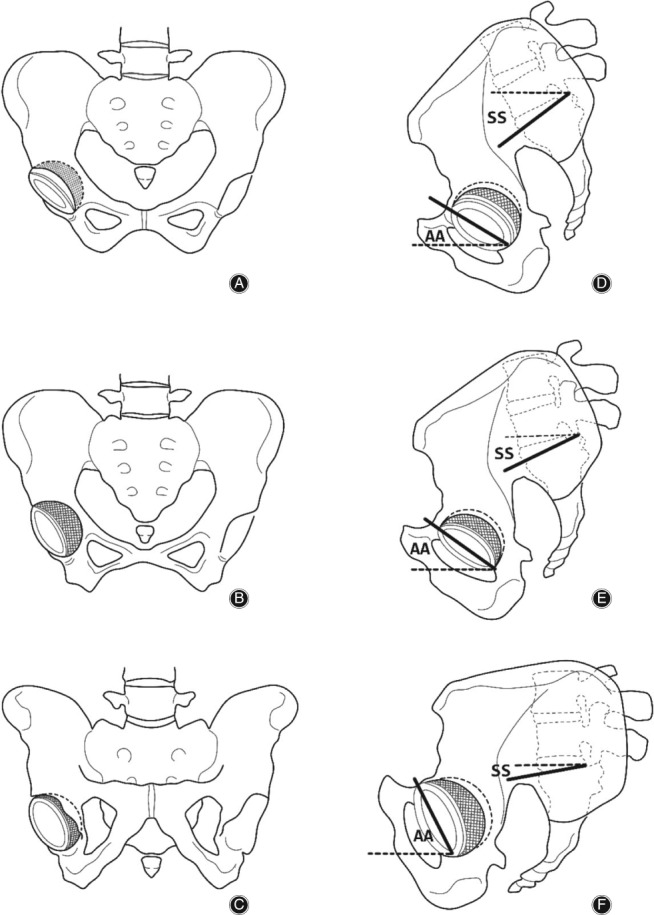
Coronal and sagittal views of the pelvis with the patient supine, standing, and sitting. AA, acetabular anteversion; ST, sacral slope. (A–C) Coronal view of the pelvis, showing coronal pelvic tilt and acetabular anteversion. From supine to standing, and to siting, pelvis is gradually tilted backwards and acetabular anteversion is gradually increasing. (D–F) Sagittal view of the pelvis, showing sagittal pelvic tilt and acetabular anteversion. From supine to standing, and to siting, pelvis is gradually tilted backwards and acetabular anteversion is also gradually increasing.

Studies have shown that pelvic tilt changes with body position^44^. Konishi *et al*.^45^ demonstrated a difference of 5° in the APP between supine and standing positions. Lazennec *et al*. reported that the pelvis leans backward by 14.5° from the standing position to the sitting position^46^. Subsequently, the group reported that SS is 46.5°, 35.0°, and 20.5° in the supine, standing, and sitting positions, respectively^34^. Chevillotte *et al*.^47^ considered that SS was 41°, 37.1°, and 11.3° in the supine, standing, and sitting positions, respectively. The magnitude of the posterior pelvic tilt increases gradually, and the pelvic tilt varies from the anterior pelvic tilt to posterior pelvic tilt at 20° from the supine position to the standing position and from the standing position to the sitting position^48^, ^49^(Fig. 4).

As the position changes, the pelvic tilt of individuals differs greatly. Sato *et al*.^50^ showed that the prosthesis would be unstable in the front if posterior pelvic tilt is >20°. Nishihara *et al*.^51^ considered that the posterior pelvic tilt is ≤10° from the supine position to the standing position in 90% of the patients, which is ≥25° from the supine position to the sitting position. Based on the degree of pelvic tilt from the supine position to the standing position, and from the supine position to the sitting position, the pelvic tilt is divided into four types. Type I (normal; 84%): the posterior pelvic tilt is ≤10° from the supine position to the standing position, or the posterior pelvic tilt is ≥20° from the supine to the sitting position. Type II (extension shift; 9%): the posterior pelvic tilt is >10° from the supine position to the standing position or >20° from the supine to the sitting position. Type III (flexion shift; 7%): the posterior pelvic tilt is ≤10° from the supine position to the standing position and is <20° from the supine position to the sitting position. Type IV (reduced 1%): the posterior pelvic tilt is >10° from the supine to the standing position and <20° from the supine to the sitting position. Kanawade *et al*.^52^ analyzed the mobility of the pelvic tilt in 85 patients from the standing to the sitting position, and the pelvic mobility was divided into three types. Type I was fixed pelvis (19%) with the mobility of <20°. Type II was pelvis with normal mobility (67%) with the mobility of 20°–35°. Type III was pelvis of high mobility (14%) with the mobility of >35°. Langston *et al*.^53^ speculated that the change in the sagittal pelvic tilt >13° is defined as poor pelvic activity as it can cause a 10° change in the acetabular anteversion, thereby possibly placing the acetabular prosthesis outside the safety zone. Moreover, limited lumbar flexion, posterior pelvic tilt in standing position, and elderly female patients are posed as risk factors for adverse pelvic activities.

## Significance of Sagittal Spinopelvic Tilt in the Preoperative Design of THA

The acetabular anteversion directly affects the stability of the joint and the life of the prosthesis after THA. The optimal acetabular anteversion remains controversial. Charnley *et al*.^54^ recommended that acetabular anteversion should be 0°, Coventry *et al*.^55^ recommended that it should be 15°, and Harris *et al*. recommended that it should be 20°^48^. In 1978, Lewinnek *et al*.^49^ proposed the concept of the “safe zone” of acetabular prosthesis, which is defined as anteversion in acetabular prosthesis of 15° ± 10° and the abduction angle of 40° ± 10°. The postoperative dislocation rate is 1.5% if the position of acetabular prosthesis is in the “safe zone,” and increases to 61% if the position is outside the “safe zone.” Since then, the concept of the “safe zone” has been widely applied in clinical practice.

**Figure 5:**
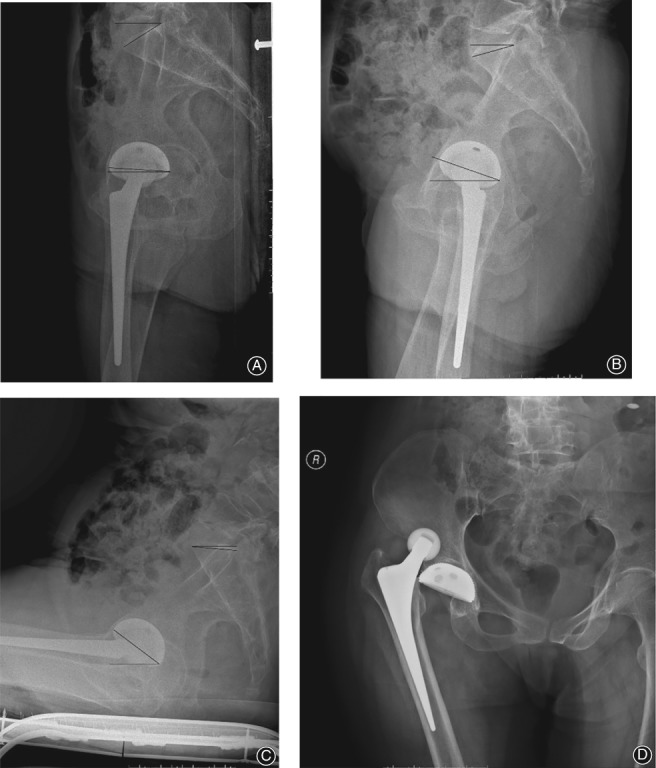
(A) Pelvic lateral radiograph in supine position, SS is 28°, acetabular anteversion is 5°. (B) Pelvic lateral radiograph in standing position, SS is 14°, acetabular anteversion is 19°. (C) Pelvic lateral radiograph in sitting position, SS is −10°, acetabular anteversion is 44°. (D) Dislocation of the hip occurs when the patient flexes the hip joint excessively in the sitting position.

Most surgeons did not fully consider the effects of changes in pelvic tilt when determining acetabular anteversion in THA. The study by Wang *et al*.^56^ showed that PT is increased in the standing position for patients with ankylosing spondylitis, and the pelvis is tilted backward. After THA, 19.2% of anteversion and 11.5% of the abduction angles for the acetabular prosthesis are outside the range of the “safe zone”; however, these follow‐up cases do not a present postoperative dislocation. Kobayashi *et al*.^57^ reported three cases with hip‐joint dislocation, and all the patients suffered from an increase in acetabular anteversion due to thoracolumbar kyphosis and excessive posterior pelvic tilt at standing; thus, dislocation would occur only by a slight abuse. After the operation, from the supine position to the standing position, the pelvis is tilted backward extremely, and the acetabular anteversion is extremely large, leading to anterior dislocation of the hip joint. The pelvic tilt changes as the altered position challenges the concept of the “safe zone” of the conventional acetabular prosthesis (Fig. 5).

The phenomenon that the acetabular anteversion changes as the pelvic tilt changes is a new challenge to the traditional “safe zone” concept. Lazennec *et al*. speculated that the “safe zone” of the acetabular prosthesis is determined in the supine position; however, most cases of postoperative dislocation occur in the standing and sitting positions, rendering the stability of the prosthesis under functional positions as crucial^58^, ^59^. McCollum *et al*.^60^ suggested that the ideal anteversion should be 20°–40° based on the APP plane. Nishihara *et al*.^51^ considered that if the acetabular anteversion is ≥20° in the supine position, then the hip‐joint prosthesis is relatively stable in the sitting position. Shon *et al*.^61^ reported an individual case who developed dislocation after THA. As can be seen, the SS in this case is 49°, and the acetabular anteversion is 26° in the supine position. On the other hand, SS is 16°, and the acetabular anteversion is 60° in the standing position. The pelvic tilt caused by postural changes must be used as an indicator of preoperative design, especially for patients with high pelvic mobility.

If the patient has high pelvic mobility, an excessive acetabular anteversion increases the risk of anterior dislocation of the hip. If the patient's pelvic mobility is low, the acetabular anteversion angle is too small to increase the risk of posterior dislocation of the hip. Kanawade *et al*.^52^ demonstrated that the posterior pelvic tilt was greater in the sitting position as compared to the supine and standing positions, and the acetabular anteversion is maximal. Moreover, anteversion >25 is a risk factor for the dislocation of patients with high pelvic mobility. The systematic review by Riviere *et al*. includes 12 articles, which has demonstrated that high pelvic mobility is a risk factor for the sign of prosthetic impingement and dislocation after THA^62^. Nonetheless, the high pelvic mobility causes an increase in acetabular anteversion from the supine position to the standing or sitting position. Furthermore, the pad wear increases, and also the anterior dislocation of the prosthesis would occur. However, Esposito *et al*.^63^ collected the lateral pelvic radiograph in the standing and the sitting positions from 1000 patients after THA. Among them, 12 patients had a postoperative dislocation, and only 1/12 cases exhibited a change in the pelvic tilt of 27° from the standing position to the sitting position, while the remaining cases have a change in pelvic tilt of 9°. Esposito *et al*. proposed that low pelvic mobility from the standing to the sitting position is a risk factor for the dislocation after THA. In patients with low pelvic activity, the acetabular anteversion angle is small during the operation, and the posterior hip instability or posterior dislocation might occur during sitting. In patients with high pelvic mobility, the acetabular anteversion angle is large during the operation, and the hip joint is unstable or dislocated when the hip joint is overextended.

Satisfactory position of acetabular prosthesis can be achieved by including changes in the pelvic tilt into preoperative planning of THA. Pierrepont *et al*.^64^ demonstrated that the activity range of sagittal pelvic plane should be evaluated before THA to obtain a suitable position of acetabular prosthesis under functional positions. Kyo *et al*.^65^ suggested imaging examination of the spine before THA. The study speculated that kyphosis would lead to compensatory posterior pelvic tilt, which affects the position of the acetabular prosthesis. Babisch *et al*.^66^ stimulated changes in the pelvic tilt in the supine and standing positions using computer software that combined the CT and X‐ray data in the preoperative planning of THA. During the operation, the direction of acetabular implantation was adjusted using the navigation technique. After the operation, the position of the acetabular prosthesis was examined by CT, indicating that 99% of the acetabular is within the target range and no case reported any dislocation. Ochi *et al*.^67^ considered that the preoperative spinopelvic parameters are correlated with the clinical efficacies after THA. Moreover, patients with anterior pelvic tilt before the operation might correct the sagittal spinopelvic force line through THA.

In summary, the changes in the acetabular anteversion caused by pelvic tilt are vital for patients with THA, which might affect the stability and life of the prosthesis. The posterior pelvic tilt leads to an increase in acetabular anteversion, while the anterior tilt leads to a decrease in acetabular anteversion. For patients with high pelvic mobility, the acetabular anteversion should be reduced appropriately in order to reduce the risk of anterior dislocation in the standing and sitting positions. In the case of patients with fixed pelvis, the anteversion in acetabular prosthesis should be increased to ensure the stability of the rear of the prosthesis in the sitting position. Before the operation, the images of the different positions of sagittal spine and pelvis should be photographed, and the characteristics of sagittal pelvic tilt should be fully considered for a customized design of anteversion in an acetabular prosthesis for patients, which is beneficial in obtaining a stable hip prosthesis and reducing the wear of the prosthesis.

### *Conclusion*

For most patients, the changes in pelvic tilt caused by factors such as body positions are not significant, and their effects on acetabular prosthesis are also relatively small; however, the individualized differences in pelvic tilt caused by spinal diseases are large. In the case of patients with severe spinal deformity or those with high sagittal pelvic mobility, the effect of pelvic tilt on the position of the acetabular prosthesis should be considered to obtain joint stability under the supine, standing, and sitting positions, thereby reducing the probability of wear, as well as the dislocation and looseness of the prosthesis. Thus, it is recommended to have lumbar and pelvic lateral radiographs in the supine, standing, and sitting positions before surgery. An individualized design of anteversion in acetabular prosthesis is fabricated by combining with factors such as spinal diseases, history of spinal surgery, spinopelvic mobility, and the age of the patient.
